# Melatonin and Vascular Function

**DOI:** 10.3390/antiox13060747

**Published:** 2024-06-20

**Authors:** Leandro Mendes, Marcelo Queiroz, Cristina M. Sena

**Affiliations:** Institute of Physiology, iCBR, Faculty of Medicine, University of Coimbra, 3000-548 Coimbra, Portugal

**Keywords:** N-acetyl-5-methoxytrypamine, immune-modulatory, anti-inflammatory, antioxidant hormone, vascular biology

## Abstract

The indolamine hormone melatonin, also known as N-acetyl-5-methoxytrypamine, is frequently associated with circadian rhythm regulation. Light can suppress melatonin secretion, and photoperiod regulates melatonin levels by promoting its production and secretion at night in response to darkness. This hormone is becoming more and more understood for its functions as an immune-modulatory, anti-inflammatory, and antioxidant hormone. Melatonin may have a major effect on several diabetes-related disturbances, such as hormonal imbalances, oxidative stress, sleep disturbances, and mood disorders, according to recent research. This has raised interest in investigating the possible therapeutic advantages of melatonin in the treatment of diabetic complications. In addition, several studies have described that melatonin has been linked to the development of diabetes, cancer, Alzheimer’s disease, immune system disorders, and heart diseases. In this review, we will highlight some of the functions of melatonin regarding vascular biology.

## 1. Introduction

**Figure 1:**
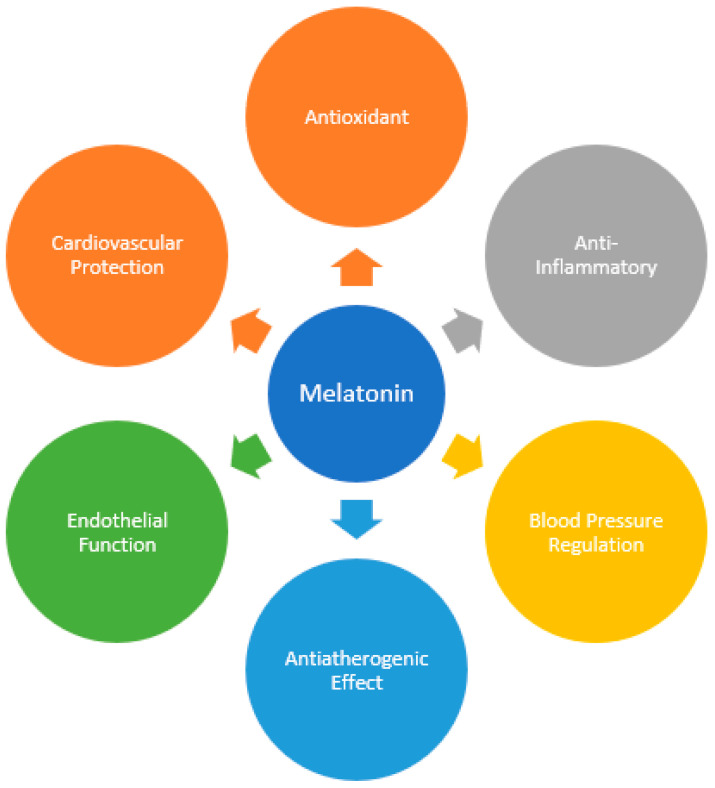
The beneficial effects of melatonin on cardiovascular health.

Melatonin, N-acetyl-5-methoxytryptamine, is an indole hormone synthesized from the essential amino acid tryptophan [1]. It is the main hormone secreted by the pineal gland and plays important roles in regulating the circadian rhythm [2]. Despite its important functions in the pineal gland, melatonin is also produced in a number of other organs, such as the retina, gut mucosa, liver, kidney, pancreas, bone marrow, heart, and endothelial cells [3,4]. Melatonin synthesis seems to occur in the mitochondria within cells [5]. Numerous other functions are associated with melatonin, namely as an antioxidant, radical scavenger, anti-inflammatory, blood pressure regulator, and immunomodulator (Figure 1) [6].

The comprehension of the endothelium has evolved significantly over time. Initially seen as inert barrier, it is now understood to be a dynamic and complex organ with multifaceted functions in various physiological processes, including vascular tone regulation, fluid and solute exchange, hemostasis, coagulation, and inflammatory responses [7,8,9]. Diseases such as diabetes, hypertension, and atherosclerosis, among others, are associated with endothelial dysfunction and other major changes in the vascular function [8].

Given the impact of cardiovascular and metabolic disorders on vascular health, it is crucial to investigate the strategies aimed at restoring endothelial function. This entails exploring mechanisms and molecules capable of enhancing endothelial function, such as boosting nitric oxide (NO) production, diminishing the generation of free radicals and vasoconstrictors, and mitigating inflammation [9]. The current understanding of the role of melatonin in vascular biology highlights its potential as a modulator of vascular function and a protector against vascular diseases. Through the mechanisms involving vasodilation, antioxidant activity, anti-inflammatory effects, blood pressure regulation, protection against ischemia-reperfusion injury, and lipid metabolism modulation, melatonin contributes significantly to cardiovascular health. Ongoing research continues to unravel its complex interactions and therapeutic potential in the vascular system. This review aims to establish a connection between the effects of melatonin—a hormone known for its antioxidant and anti-inflammatory properties—and the beneficial protection in vascular function.

## 2. Melatonin: A Regulator of Circadian Rhythms

**Figure 2:**
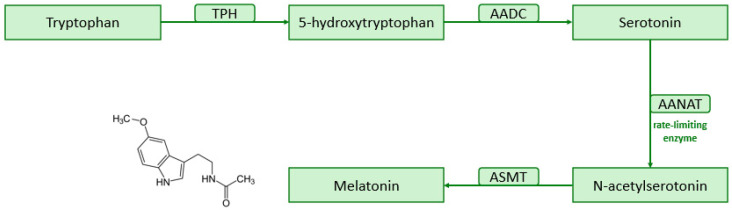
Pathway of melatonin synthesis. AADC, aromatic amino acid decarboxylase; AANAT, arylalkylamine N-acetyltransferase; ASMT, N-acetylserotonin O-methyltransferase; TPH, tryptophan hydroxylase.

The melatonin biosynthetic process begins with the amino acid tryptophan, acquired through the diet, and occurs in four essential steps [1,10]. First, the hydroxylation of tryptophan into 5-hydroxytryptophan by the enzyme tryptophan hydroxylase (TPH). Then, the decarboxylation of 5-hydroxytryptophan, in the presence of the aromatic amino acid decarboxylase (AADC), generating 5-hydroxytryptamine (serotonin). Serotonin then undergoes acetylation by serotonin N-acetyltransferase (SNAT or AANAT, the acronym for arylalkylamine N-acetyltransferase—the rate-limiting enzyme), converting into N-acetylserotonin. Finally, melatonin is produced through the methylation of N-acetylserotonin by N-acetylserotonin O-methyltransferase (ASMT, previously identified as hydroxyindole-O-methyltransferase or HIOMT) [2,10,11,12,13]. The melatonin synthesis pathway is schematized in Figure 2.

Melatonin levels in the serum exhibit significant variations across different age groups [6,13]. During infancy, the melatonin secretion is minimal, with levels remaining low until around 3 months of age. From infancy through adolescence, the melatonin levels progressively rise, reaching a plateau, before gradually decreasing as individuals enter their late twenties to their fifties. Elderly people produce residual levels of melatonin [13,14]. Melatonin levels in early life are crucial for vascular health, promoting vasodilation, reducing oxidative stress, and supporting endothelial function. However, as melatonin levels decline in adulthood [15], this may lead to increased oxidative stress, inflammation, and endothelial dysfunction, increasing the risk of hypertension, atherosclerosis, and other cardiovascular diseases. The elderly are more prone to oxidative stress and inflammation, contributing to the progression of vascular diseases [16,17].

Melatonin production and release are stimulated by darkness and suppressed by light. In humans, melatonin secretion initiates shortly after sunset, peaks in the middle of the night, and gradually diminishes during the latter half of the night [18]. Approximately 80% of melatonin is synthesized during the nighttime, resulting in serum levels ranging from 80 to 120 pg/mL. Conversely, during the daylight hours, serum concentrations remain low, typically between 10 and 20 pg/mL [2,18].

Melatonin synthesis occurs, as previously outlined, not only in the pineal gland but also in other tissues. The complex signaling cascade that results in the production of melatonin begins with the recognition of light, which takes place in specific retinal cells: the intrinsically photosensitive retinal ganglion cells (ipRGCs) [4]. These retinal cells send the light information to the suprachiasmatic nucleus (SCN) via the retinohypothalamic tract. From there, a neural pathway begins from the SCN to the paraventricular nucleus, traversing through the brainstem, the spinal cord, and ultimately reaching the pineal gland via the superior cervical ganglion (SCG) [4,19,20,21]. Norepinephrine (NE) is released into the synapse by adrenergic neurons projecting from the SCG. Upon release, NE binds to both beta-1 (β1) and alpha-1 (α1) receptors located on the cell membrane of the pinealocytes. On the one hand, NE induces the activation of the α1-adrenergic receptors, thereby resulting in an increase in the cytoplasmic calcium ion concentrations. On the other hand, the stimulation of the β1-adrenergic receptors by NE initiates a signaling cascade, activating adenylate cyclase (AC) to elevate cytoplasmic cyclic adenosine-3,5-monophosphate (cAMP). This increase in cAMP levels triggers the activation of cAMP-dependent protein kinase A (PKA), which stimulates the production of AA-NAT (by stimulate the transcription of AA-NAT RNA), promoting melatonin biosynthesis [3,4,19,20,21].

### 2.1. Insights from Research in Non-Pineal Tissues

The synthesis of melatonin in non-pineal tissues underscores its diverse physiological roles beyond the regulation of sleep. Tryptophan is converted into melatonin in the pineal gland and by practically every organ in the body because the mitochondria are involved in the process [22]. Indeed, the mitochondria are crucial for melatonin synthesis, metabolism, and activity [5]. It has been demonstrated that the mitochondria, as opposed to the circadian (light/dark) cycle, stimulate melatonin production in response to cellular needs. In comparison with the blood, the mitochondria have a higher concentration of melatonin. This is because the mitochondria with electron transport chains have higher requirements for an antioxidant pool [23,24,25].

Melatonin contributes to tissue-specific functions, including protection against oxidative stress, the modulation of immune responses, and the regulation of circadian rhythms [6]. Notably, elevated melatonin levels are beneficial to health and the aging process [26]. The gastrointestinal tract is a major extrapineal source of melatonin. The enterochromaffin cells in the gut can produce melatonin independently of the pineal gland. This melatonin is involved in regulating gut motility, modulating immune responses, and protecting the gastrointestinal mucosa from damage caused by oxidative stress and inflammation [27].

Melatonin production in immune cells, such as lymphocytes and macrophages, suggests its role in modulating immune responses [28,29]. Recent research has focused on how the melatonin produced by these cells influences inflammation, autoimmunity, and the overall immune response, highlighting its potential in treating inflammatory and autoimmune diseases [28,29,30]. In addition, melatonin synthesis in bone marrow cells has been implicated in hematopoiesis and the regulation of circadian rhythms in bone marrow-derived cells. This area of research is expanding our understanding of the role of melatonin in bone health and its potential therapeutic applications in hematological disorders [31].

Understanding these mechanisms opens new avenues for therapeutic interventions, targeting various conditions ranging from gastrointestinal disorders to immune-related pathologies including vascular diseases.

### 2.2. Melatonin Receptors

**Figure 3:**
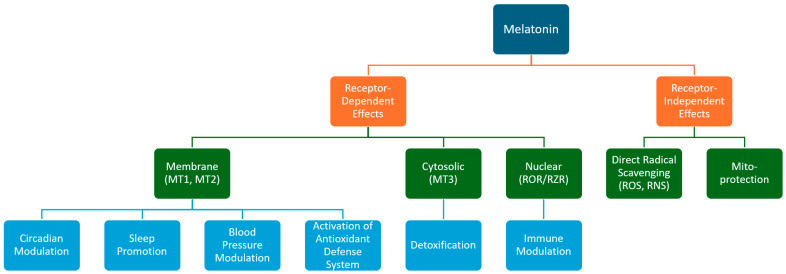
Schematic figure of the receptor-dependent and -independent effects of melatonin. MT 1,2,3, melatonin receptors; ROR/RZR, retinoid orphan receptors or retinoid Z receptors; ROS, reactive oxygen species; RNS, reactive nitrogen species.

The functions of melatonin may occur due to interactions with receptors and targets, as well as through receptor-independent mechanisms (Figure 3) [32]. The melatonin receptors MT1 and MT2 are transmembrane receptors, belonging to the class of G-protein-coupled receptors. The MT1 receptor is encoded on chromosome 4 and consists of 350 amino acids, while the MT2 receptor is encoded on chromosome 11 and consists of 362 amino acids [33,34,35]. Melatonin binds and activates the MT1/MT2 receptors, resulting in the inhibition of the AC/cAMP/PKA/CREB (cAMP response element-binding protein) and GC/cGMP/PKG signaling pathways, which reduces cAMP and cyclic guanosine-3,5-monophosphate (cGMP) levels, leading to the activation of calcium signaling by calmodulin kinases and protein kinase C. This allows the melatonin to regulate hormone synthesis and activate the antioxidant defense system [11,32,35,36]. Additionally, the melatonin activation of the MT1/MT2 receptors initiates other signaling pathways, such mitogen-activated protein kinases and extracellular-signal-regulated kinase (ERK1/2). These pathways play a role in several regulatory processes, including the cell responses to various injuries and chronobiological regulation [32,35,36,37].

Intracellularly, melatonin has the capacity to interact with the MT3 receptor, identified as a cytosolic receptor with a minimal affinity for melatonin. Functioning as a quinone reductase 2, it possesses the ability to neutralize free radicals [11,32,36]. Melatonin can also bind to nuclear receptors known as retinoid orphan receptors or retinoid Z receptors, which play roles in immune modulation and the regulation of antioxidant enzymes; however, a consensus has not yet been reached [11,36,38]. The receptor-independent action of melatonin consists of its direct antioxidant capacity, its effects on different protein targets, and its mitochondria protection (Figure 3) [36].

The important role played by melatonin in the sleep–wake cycle is due to the presence of the MT1 and MT2 receptors in the SCN (the circadian clock) [2,21]. Although the production and release of melatonin occurs in the pineal gland, under SCN regulation, this molecule feeds back to the SCN through the MT1 and MT2 receptors, to reduce neuronal firing or induce a change in the circadian phase. Melatonin is also capable of affecting the expression of the clock genes in the SCN [1,21,39].

Melatonin receptors are distributed across both the central and peripheral tissues, including the cardiovascular system (the peripheral blood vessels, aorta, and heart). The physiological effects of melatonin depend on the localization and types of the melatonin receptors [2,34]. The MT1 and MT2 receptors are integral to the cardiovascular protective effects of melatonin. Through mechanisms involving the regulation of vascular tone, anti-inflammatory and antioxidant actions, circadian regulation, and antithrombotic effects, these receptors help to maintain cardiovascular health and protect against various cardiovascular diseases.

#### 2.2.1. MT1 Receptors

MT1 receptors are involved in the regulation of vascular tone. In humans, the activation of MT1 receptors on vascular smooth muscle cells leads to vasoconstriction [40,41]. MT1 receptors enhance the antioxidant defenses by upregulating the expression of antioxidant enzymes such as superoxide dismutase (SOD) and glutathione peroxidase. By reducing the oxidative stress, MT1 receptors protect the vascular endothelium from the damage caused by reactive oxygen species (ROS), thereby preserving endothelial function and preventing vascular diseases [41].

MT1 receptors also modulate immune responses and reduce inflammation [28]. Activation of these receptors can inhibit the release of pro-inflammatory cytokines and suppress the activation of inflammatory pathways such as nuclear factor-κB (NF-κB). Reduced inflammation in the vascular endothelium helps to prevent endothelial dysfunction and atherosclerosis, which are critical in maintaining cardiovascular health [7,42,43].

#### 2.2.2. MT2 Receptors

In contrast to the MT1 receptors, the activation of MT2 receptors causes vasodilation. This leads to the stimulation of NO production in the endothelial cells through the activation of endothelial nitric oxide synthase (eNOS). The increase in NO production results in vasodilation and improved blood flow, which is beneficial for maintaining optimal vascular function and reducing blood pressure [44].

In addition, MT2 receptors play a crucial role in the regulation of circadian rhythms, which include the circadian regulation of the blood pressure and heart rate. The proper functioning of circadian rhythms helps in maintaining cardiovascular health by preventing circadian disruption-related cardiovascular events such as hypertension and heart attacks [16,35].

MT2 receptor activation has also been associated with antithrombotic effects. These receptors can inhibit platelet aggregation and reduce the risk of thrombosis. By preventing excessive platelet aggregation, MT2 receptors help in reducing the risk of thrombotic events such as stroke and myocardial infarction [45].

#### 2.2.3. Combined Effects of MT1 and MT2 Receptors

Melatonin, through its receptors, reduces the progression of atherosclerosis by preventing endothelial dysfunction, reducing oxidative stress, and inhibiting inflammation. These effects are mediated by the coordinated action of the MT1 and MT2 receptors [46]. In the context of ischemia-reperfusion injury, melatonin receptor activation has shown protective effects on the heart. Both MT1 and MT2 receptors help in reducing myocardial damage by mitigating oxidative stress, inflammation, and apoptosis during reperfusion [47].

Understanding the roles of the MT1 and MT2 receptors in cardiovascular protection opens up potential therapeutic avenues. Melatonin or selective melatonin receptor agonists could be used to treat hypertension, prevent atherosclerosis, and protect against ischemic heart diseases [48]. The variations in melatonin receptor expression and function among individuals suggest that personalized approaches to melatonin-based therapies could optimize cardiovascular outcomes.

## 3. Antioxidant Properties of Melatonin

Free radicals are continually produced in normal aerobic functions and are involved in several biological processes [49]. ROS comprise various molecules, from the superoxide anion radical (O_2_^•−^), primarily generated within the cytosol, mitochondria, and endoplasmic reticulum, to hydrogen peroxide (H_2_O_2_), which is synthesized in peroxisomes, and the hydroxyl radical (^•^OH) and singlet oxygen (^1^O_2_), which are also highly reactive species. Alongside ROS, reactive nitrogen species can contribute to cellular damage, exemplified by nitric oxide (^•^NO) and generated by NO-synthases. Nitric oxide reacts with O_2_^•−^ to produce peroxynitrite (ONOO^−^), a potent oxidative and nitrosative agent [50,51]. The concentration of these substances is strongly controlled by the different antioxidants present in cells, considering that they also serve as second messengers for different cellular processes (as in the case of nitric oxide) [51]. The disturbances that occur in the balance between the concentration of radical species and antioxidant defenses result in a state of oxidative stress, which can cause cellular damage, with a loss of function and integrity [52].

Oxidative stress is associated with various cardiovascular pathologies, such as atherosclerosis and hypertension, through different mechanisms, namely the promotion of inflammation and endothelial dysfunction [53,54].

The antioxidant power of melatonin has been described in the literature for several years [55]. This molecule can exert its antioxidant activity directly, through radical scavenging, or indirectly, through the activation of antioxidant enzymes and the inhibition of pro-oxidant enzymes [36]. The intense antioxidant activity of melatonin is due, on the one hand, to its high intracellular concentration in the mitochondria, which allows for better functioning of the respiratory chain, with less generation of free radicals (radical avoidance) [23,24]. On the other hand, the intermediate compounds (cyclic 3-hydroxymelatonin, N1-acetyl-N2-formyl-5-methoxykynuramine, and N-acetyl-5-methoxykynuramine), produced through the reaction of melatonin with different radical species, are also strong antioxidants [56]. In this way, melatonin can neutralize up to ten ROS, when traditional antioxidants typically neutralize one ROS [24,36]. Melatonin has also been shown to be capable of inhibiting metal-induced oxidation, in processes such as lipid peroxidation, through the formation of chelates with different transition metals [57,58]. Melatonin also has the ability to activate different enzymes, responsible for catalyzing antioxidant reactions, eliminating free radicals. By binding to the MT1 and MT2 receptors, melatonin can stimulate the expression and activity of enzymes such as superoxide dismutase (SOD—which reduces the superoxide radical O_2_^•−^ to H_2_O_2_), catalase (which decomposes hydrogen peroxide into water and oxygen), and glutathione peroxidase and glutathione reductase (which catalyze the GSSH/GSH reaction that is responsible for activating the antioxidant activity of glutathione, which decomposes hydrogen peroxide). In terms of inhibiting the pro-oxidant enzymes, melatonin is responsible for suppressing lipoxygenase activity [23,24,51,59,60].

Pimenta and co-workers conducted experiments to evaluate the effect of melatonin on the production of ROS and vascular dysfunction induced by cyclophosphamide and reported that melatonin demonstrated vasoprotective effects. It inhibits NADPH oxidase activity, enhances SOD activity, and elevates reduced glutathione (GSH) levels, while also reducing the production of pro-inflammatory cytokines [61]. Ren et al. reported that melatonin had a protective effect against the harmful effects of oxidative stress in the diabetic aorta, obtained using an STZ-induced diabetic animal model and vascular smooth muscle cells (VSMCs). In this study, melatonin, in addition to demonstrating its antioxidant role, managed to activate the Notch1 signaling pathway and reduce the expression of pro-apoptotic proteins [62].

### 3.1. Comparison of Melatonin with Other Antioxidants

The antioxidant properties of melatonin play a crucial role in protecting against oxidative stress and related diseases. To highlight the unique benefits of melatonin, it is useful to compare it with other well-known antioxidants, such as vitamin C and vitamin E, glutathione, and coenzyme Q10. This comparison can elucidate the distinctive features of melatonin and the advantages in the context of antioxidant defense and cardiovascular protection.

#### 3.1.1. Melatonin vs. Vitamin C

Vitamin C primarily acts in the aqueous compartments of the cell (cytosol and plasma). It neutralizes free radicals by donating electrons and is effective in reducing oxidative stress [63]. Melatonin functions in both aqueous and lipid environments. It directly scavenges a wide range of reactive oxygen and nitrogen species (ROS/RNS) and upregulates antioxidant enzymes [23,24,55]. Vitamin C is limited to the aqueous phases and needs transporters for cellular uptake [64]. Melatonin is highly lipophilic, allowing it to cross all the cellular membranes, including the blood–brain barrier, providing widespread protection [65].

Vitamin C can be regenerated from its oxidized form (dehydroascorbate) by cellular reductants such as glutathione [64]. Unlike vitamin C, melatonin is not regenerated after scavenging free radicals. Its metabolites continue to exhibit antioxidant properties, leading to a cascade of antioxidant actions [56].

#### 3.1.2. Melatonin vs. Vitamin E

Vitamin E is primarily an antioxidant with limited additional cellular effects. It functions primarily in lipid environments, protecting cell membranes from lipid peroxidation by donating a hydrogen atom to the lipid radicals [64]. Melatonin provides antioxidant protection in both the lipid and aqueous phases and also protects against protein oxidation and DNA damage [66].

Vitamin E requires co-antioxidants such as vitamin C to regenerate its active form after neutralizing free radicals, while melatonin does not rely on regeneration. Its metabolites (e.g., N1-acetyl-N2-formyl-5-methoxykynuramine) continue to exhibit antioxidant activity. Besides being an antioxidant, it has anti-inflammatory, circadian rhythm-regulating, and immune-modulating properties [25,28,67].

#### 3.1.3. Melatonin vs. Coenzyme Q10 (Ubiquinone)

Coenzyme Q10 functions within the mitochondrial membrane, participating in electron transport and reducing oxidative damage by acting as a lipid-soluble antioxidant [68]. Melatonin protects the mitochondria from oxidative stress and enhances mitochondrial function, directly scavenging radicals and reducing mitochondrial ROS production [69].

Coenzyme Q10 is concentrated within the mitochondria while melatonin is widely distributed, including in the mitochondria, cytosol, and cellular membranes. Coenzyme Q10 is directly involved in ATP production and cellular energy metabolism [69]. Melatonin indirectly supports energy metabolism by protecting the mitochondrial integrity and function [22].

#### 3.1.4. Melatonin vs. Glutathione

Glutathione is a tripeptide, present in high concentrations in cells (primarily in the cytosol, mitochondria, and nucleus), which acts as a major intracellular antioxidant, directly neutralizing the free radicals and regenerating other antioxidants. Glutathione levels can be depleted under conditions of severe oxidative stress [70]. Melatonin can work synergistically with glutathione by increasing its synthesis and activity. In contrast to glutathione, melatonin is present in lower concentrations but is more widely distributed across all cellular compartments. Melatonin stimulates the activity of the enzymes involved in glutathione regeneration and synthesis, helping to maintain glutathione levels [71].

Noteworthy, the unique benefits of melatonin, including its broad-spectrum antioxidant and anti-inflammatory activities, metabolite efficacy, circadian regulation, and multi-compartment distribution, distinguish it from other antioxidants. These features make melatonin particularly effective in providing comprehensive cardiovascular protection and enhancing overall health. Incorporating melatonin in therapeutic strategies offers a promising approach to mitigating oxidative stress and related vascular diseases across different age groups.

## 4. Anti-Inflammatory Effects

Inflammation is a normal response of organisms to recover from tissue damage or infections; however, chronic and uncontrolled inflammation can cause extensive tissue damage [72]. Inflammation and oxidative stress are closely linked, being preponderant in several cardiovascular diseases [53,54]. Diseases such as obesity and diabetes increase the cardiovascular risk, due to an increase in ROS and inflammation, and are considered chronic diseases with a low level of inflammation [72,73].

In cardiovascular inflammation, there is frequently an elevation in inflammatory cytokines such as interleukin-1β (IL-1β), interleukin-6 (IL-6), interleukin-18 (IL-18), and tumor necrosis factor-α (TNFα), released by resident and infiltrating immune cells. This is accompanied by a parallel decrease in anti-inflammatory cytokines such as interleukin-4 (IL-4), interleukin-10 (IL-10), and transforming growth factor beta [72,74,75].

Patients with metabolic syndrome often experience decreased levels of potent vasodilators, such as NO, alongside increased levels of endothelin-1, a vasoconstrictor [72]. These alterations coincide with the release of pro-inflammatory cytokines. The dysregulated production of pro-inflammatory cytokines exacerbates tissue injury through mechanisms that involve leukocyte recruitment, ROS generation, mitochondrial dysfunction, fibrosis, and cell death [72,76].

In animal studies, melatonin administration has demonstrated the ability to decrease the inflammatory response, through the reduction of pro-inflammatory cytokines such as IL-1β and TNF-α and an increase in anti-inflammatory cytokine IL-4 levels in the serum. Melatonin also inhibited the expression of cyclooxygenase and inducible nitric oxide synthase and decreased the production of other inflammatory mediators such as prostanoids, leukotrienes, chemokines, and adhesion molecules [77,78,79].

In an animal model of atherosclerosis, Chen and collaborators demonstrated that melatonin exhibited an antiatherogenic effect by inhibiting the S100a9/NF-κB signaling pathway-mediated vascular inflammation. Melatonin additionally reduced atherosclerotic lesions, promoted stable phenotypic sclerotic plaques, inhibited macrophage infiltration, and suppressed the production of proinflammatory cytokines [80].

In a study involving animal models of type 2 diabetes, Yu and co-workers showed that prolonged administration of melatonin mitigated the progression of diabetic cardiomyopathy (characterized by inflammation, fibrosis, and impairing cardiac function) and lowered myocardial susceptibility to myocardial ischemia reperfusion (MI/R) injury. This effect was achieved by reducing mitochondrial fission and boosting mitochondrial biogenesis and mitophagy through the reactivation of the SIRT6 and AMPK-PGC1α-AKT signaling pathways [81].

The interaction of melatonin with common inflammatory markers highlights its potential as a powerful anti-inflammatory agent in vascular diseases. By reducing the levels of C-reactive protein (CRP), TNF-α, IL-6, IL-1β, matrix metalloproteinases, and adhesion molecules, melatonin helps to mitigate inflammation, stabilize atherosclerotic plaques, and improve endothelial function [77,78,79]. Furthermore, to reduce the inflammatory processes at the cellular level, melatonin may also downregulate NF-κB, a proinflammatory transcription factor [80], and upregulate nuclear factor erythroid 2-related factor 2 (Nrf2) [82,83], an anti-inflammatory transcription factor. Similarly, this indoleamine promotes the polarization of macrophages from a proinflammatory phenotype (M1 phenotype) to an anti-inflammatory phenotype (M2 phenotype) and stimulates the release of anti-inflammatory cytokines such as IL-4 and IL-10 [17]. Melatonin also suppresses proinflammatory events, and cyclooxygenase-2 and NLR family pyrin domain containing 3 (NLRP3) inflammasome activation [84]. It also upregulates the expression of Klotho [85], an antiaging protein with powerful antioxidant, anti-inflammatory, and antiapoptotic properties. These properties underscore the therapeutic potential of melatonin in cardiovascular protection and the management of vascular diseases. Since cardiometabolic diseases and aging [86] are typically linked to chronic proinflammatory processes—which are a result, at least partially, of reductions in endogenous melatonin secretion—all these actions together significantly contribute to the protective effect of exogenous melatonin [17].

## 5. Endothelial Function and Nitric Oxide Production

The endothelium is a layer of cells that lines the inside of the blood vessels, forming a semipermeable barrier between the blood and the surrounding tissues. It performs several essential functions that are important in cardiovascular health and vascular homeostasis, such as regulating vascular tone, and controlling blood flow and inflammatory responses [7,8]. In physiological conditions, endothelial cells carefully regulate the balance between ROS production and the scavenging activity of endogenous antioxidants. However, in certain pathophysiological states such as hyperlipidemia, ischemia-reperfusion injury, and shear stress injury, this equilibrium can be disrupted. This disturbance leads to oxidative stress, which can induce endothelial dysfunction, thus exacerbating the progression of cardiovascular diseases [7,50,87].

In endothelial cells, NO plays a crucial role in maintaining vascular homeostasis. It is generated within endothelial cells through the conversion of L-arginine to L-citrulline by the enzyme eNOS. Subsequently, NO diffuses to VSMCs, where it triggers the activation of soluble guanylate cyclase, leading to an elevation in the levels of cGMP and inducing the relaxation of VSMCs [7,88]. A decrease in NO availability, caused by either reduced NO production or increased NO degradation, is indicative of the onset of endothelial dysfunction [8].

In cardiovascular diseases, oxidative stress plays a major role in reducing eNOS activity and NO bioavailability, through reducing the tetrahydrobiopterin or L-arginine levels. This reduction can lead to eNOS uncoupling, generating superoxide instead of NO [50]. ROS also have the ability to inactivate NO through the formation of peroxynitrite, which contributes to the exacerbation of oxidative stress [50,54]. In both humans and animal models with atherosclerosis, reduced expression and activity of eNOS was demonstrated, leading to a decline in the NO production [87].

Atherosclerosis is the consequence of cholesterol build-up and chronic inflammation, in the context of a dysfunctional endothelium [46]. Melatonin exhibits favorable anti-atherosclerotic properties through several different mechanisms, such as the inhibition of the formation of endothelium-derived adhesion molecules, the reduction of fatty acid infiltration into the endothelial layer, the neutralization of free radicals, the reduction of lipid peroxidation, the inhibition of inflammatory pathways, and the prevention of electron leakage from the mitochondrial respiratory chain [46,87,89].

Wakatsuki and co-workers have demonstrated, in vitro, that melatonin offers protection against the inhibition of NO production caused by oxidized low-density lipoprotein (ox-LDL) [90]. Peng Li and collaborators have shown that melatonin effectively mitigates ox-LDL-induced damage in endothelial cells by preserving endoplasmic reticulum homeostasis, mitochondrial function, and antioxidant processes [91]. Moreover, melatonin has exhibited protective effects on the local vasculature afflicted with atherosclerotic damage, by suppressing the toll-like receptor 4 (TLR4)/NF-κB pathway, which serves as the principal regulator of inflammation [92]. Another example brings together the action of statins and melatonin and has corroborated that melatonin diminishes oxidative stress and enhances the statins’ ability to stimulate eNOS, consequently augmenting NO production and eliciting vasodilation [93].

## 6. Perivascular Adipose Tissue

Long-term melatonin treatment normalized the anticontractile effects of perivascular adipose tissue (PVAT) in mice models of accelerated aging. It was also linked to increased expressions of the vasoprotective markers, decreased oxidative stress, and reduced inflammation in PVAT [94]. In a recent study, melatonin treatment reversed the excess ROS production, restored SOD activity, and increased the NO bioavailability in obese rats, restoring the anticontractile effect of aortic PVAT [95]. These anticontractile effects of PVAT contributed to reduced blood pressure.

## 7. Blood Pressure Regulation

From a physiological standpoint, blood pressure typically stays within a normal range due to a delicate equilibrium between the factors that may elevate blood pressure and those that regulate it, functioning as compensatory mechanisms. When this balance is disrupted, hypertension occurs [96]. The pathophysiology of hypertension entails the overproduction of the vasoconstrictor agents and oxidative stress, culminating in endothelial dysfunction, which may lead to alterations in both the macro- and micro-structure of the vasculature. Indeed, arterial stiffening is a primary consequence of chronic stress, induced by dyslipidemia, aging, and elevated blood pressure [50,53,96].

**Figure 4:**
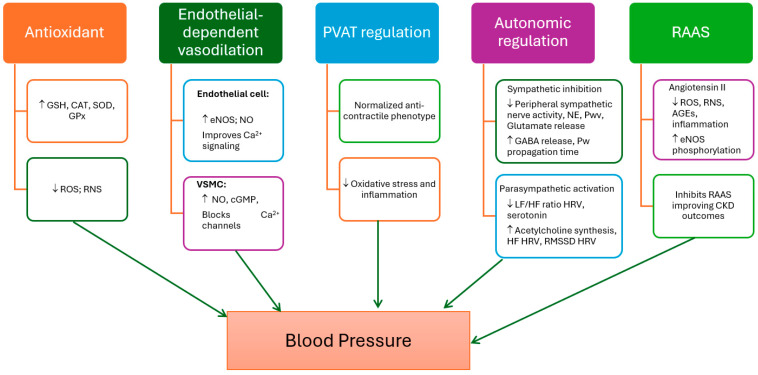
The role of melatonin in blood pressure regulation: mechanisms involved. AGE, advanced glycation end product; CAT, catalase; cGMP, cyclic guanosine-3,5-monophosphate; CKD, chronic kidney disease; eNOS, endothelial nitric oxide synthase; GPx, glutathione peroxidase; GSH, reduced glutathione; HF, high frequency; HRV, heart rate variability; LF, low frequency; NE, norepinephrine; NO, nitric oxide; RAAS, renin-angiotensin-aldosterone system; ROS, reactive oxygen species; RMSSD, Root Mean Square of the Successive Differences; RNS, reactive nitrogen species; SOD, superoxide dismutase.

Reduced melatonin secretion and production are closely associated with the onset of nocturnal and essential hypertension. Consequently, melatonin is starting to become regarded as a possible adjunct anti-hypertensive agent. A correlation has been established in elderly individuals between reduced nocturnal melatonin secretion and hypertension [97]. The positive effects of melatonin on hypertension may occur due to different mechanisms including its antioxidant properties and endothelial-dependent vasodilation actions [98,99,100]. In addition, melatonin also influences the regulation of blood pressure through its effects on the autonomic nervous system and the renin–angiotensin system [101,102,103]. Several studies have suggested that melatonin may help to lower blood pressure, which is a key risk factor for many vascular diseases, including stroke and heart disease [104,105], although some contradictory reports have also been described [88,89]. A summary of the main mechanisms is presented in Figure 4.

The long-term administration of melatonin (for a duration of 2 months) has been shown to elevate catalase activity, thereby decreasing the oxidative stress indicators and mitigating hypertension in individuals with metabolic syndrome [104].

Melatonin has been observed to reduce NF-κB-induced oxidative stress and inflammation in spontaneously hypertensive rats [106]. In a rat model of pulmonary hypertension, melatonin therapy ameliorated right ventricular hypertrophy and dysfunction, while also diminishing interstitial fibrosis and oxidative stress. Remarkably, melatonin supplementation decreased nocturnal hypertension, blood pressure, platelet aggregation, and circulating catecholamines [77].

In humans, studies have also revealed a significant influence of melatonin that is suggestive of vasodilation, including reduced vascular resistance, decreased pulse wave velocities, and decreased pulsatility indices with a subsequent lowering of the blood pressure [100,107,108]. In individuals with obstructive sleep apnea, hypertension has been linked to endothelial dysfunction triggered by chronic intermittent hypoxia. Moreover, melatonin treatment in rats with chronic intermittent hypoxia has been shown to improve levels of NO, endothelial-dependent relaxation, and the expression of eNOS and antioxidant enzymes [109].

Angiotensin II (Ang II) is recognized as a vasoactive peptide in the renin–angiotensin system, playing a pivotal role in the pathogenesis of hypertension [110]. Stimulation with Ang II in human aortic endothelial cells induces an increased generation of ROS and impedes the phosphorylation of eNOS at Ser1177. In rats infused with Ang II, increased ROS production within the aortic wall and the impaired endothelial function of the aortic ring have been observed. Pretreatment with melatonin lowered the oxidative stress and restored the phosphorylation of eNOS, and the co-administration of melatonin in rats rescued the harmful effects of Ang II administration [111]. Melatonin may mitigate the oxidative damage caused by angiotensin by inhibiting the synthesis of inflammatory cytokines, advanced glycation end products, and reactive oxygen and nitrogen species. By inhibiting the renin–angiotensin II–aldosterone system, melatonin improves the course of chronic kidney disease [101].

Melatonin secretion is reduced in patients with coronary artery disease, and nocturnal urinary melatonin excretion was found to be inversely correlated with the non-dipper pattern of hypertensive disease in older hypertensive patients [112]. Administering 5 mg of melatonin daily has been shown to lower the nocturnal blood pressure in hypertensive patients and lessen age-related disruptions in their cardiovascular rhythms [105,113]. In addition, exogenous melatonin has been studied in both healthy and human patients in relation to the regulation of autonomic and blood pressure. Low doses of melatonin (1 mg) have been shown to significantly reduce the mean, diastolic, and systolic blood pressure in healthy men and women. They have also been shown to significantly lower the NE levels and the internal carotid artery pulsatility index, which is a direct indicator of vasoconstriction-related blood flow impedance [98,107,108,114]. Similar studies have found that 2 mg of oral melatonin increased the parasympathetic parameters of heart rate variability [102], decreased the supine blood pressure, and significantly reduced the supine plasma NE and dopamine levels. Finally, 3 mg of oral melatonin significantly decreased the increase in sympathetic activity, as measured using direct sympathetic measures in response to an orthostatic challenge, a maneuver linked to increased sympathetic activity [103].

## 8. Platelet Aggregation

The action of melatonin on platelet aggregation is an important aspect related to vascular health. Melatonin has antithrombotic effects with an impact on platelet aggregation and activated coagulation [115]. Melatonin can suppress the production of thromboxane, a potent platelet aggregator and vasoconstrictor. By inhibiting thromboxane synthesis, melatonin helps to maintain vascular homeostasis and prevent excessive platelet aggregation. Noteworthy, MT2 receptor activation has been associated with antithrombotic effects. These receptors can inhibit platelet aggregation and reduce the risk of thrombotic events such as stroke and myocardial infarction [45,116].

Excessive platelet aggregation is a key factor in the formation of blood clots and thrombosis, which can lead to cardiovascular events such as heart attacks and strokes. By inhibiting platelet aggregation, melatonin may reduce the risk of thrombotic events and improve cardiovascular health. Previous research showed low melatonin levels in people with type 2 diabetes, insulin resistance, and coronary artery disease [117,118,119]. In light of this, melatonin therapy may prove to be a useful tactic in the treatment of atherothrombotic disease, especially in high-risk individuals with abnormal circadian rhythms, such as shift workers. Melatonin supplementation has been shown in experimental work to ameliorate the insulin resistance resulting from internal circadian rhythm disruption [120].

Subjects with coronary artery disease secrete less melatonin at night than healthy people, as do patients with unstable angina as opposed to stable angina [113,117,121]. Due to the compromised circadian biological rhythmicity and the absence of the calming effect of melatonin on sympathetic activity, endothelial damage, platelet activation, and the vulnerability of vulnerable plaques to rupture are all caused by sympathetic activation [16,117,122]. The activation of the coagulation cascade and elevated sympathetic activity in the early morning may be responsible for the well-documented morning peaks in cardiovascular events in patients with coronary artery disease [123,124]. Furthermore, elevated sympathetic activity may impact the synthesis of plasminogen activator inhibitor-1, a critical inhibitor of fibrinolysis, which could lead to hypofibrinolysis and elevate the risk of vascular events [125].

Melatonin is believed to affect the circadian variation in platelet activity in addition to the proteins involved in coagulation. Several studies have indicated that melatonin directly affects platelet function. Melatonin has been linked to the suppression of induced and spontaneous platelet aggregation [126,127]. Furthermore, some research, but not all research, indicates that melatonin increases platelet apoptotic events [128,129]. The process of thrombogenesis may be inhibited by either of these melatonin-induced effects on platelets, but more research is necessary because the supporting data are inconsistent.

## 9. Research Findings and Inconsistencies

In a systematic review and meta-analysis of randomized controlled trials, it has been reported that treatment with exogenous melatonin has positive effects on sleep quality (assessed by the Pittsburgh Sleep Quality Index) in adults with respiratory diseases, metabolic disorders, and primary sleep disorders, but not with mental disorders, neurodegenerative diseases, and other diseases [130].

In two other meta-analyses, with the aim of studying the effect of melatonin supplementation on inflammation biomarkers, the authors showed that melatonin supplementation significantly decreased TNF-α and IL-6 levels, had a marginal effect on CRP levels, and had a large anti-inflammatory effect on IL-1, IL-6, and IL-8 [131,132].

In a study on the effects of melatonin on metabolic diseases such as diabetes, supplementation with melatonin reduced fasting blood glucose, glycated hemoglobin, and insulin resistance compared with placebo, in a meta-analysis performed by Delpino et al. [133].

Supplementation with melatonin in controlling blood pressure had some promising results, with reports that melatonin significantly reduced the nocturnal blood pressure [134,135]; however, there were also contradictory reports, which point to the lack of significant improvements when compared with the placebo group [67,136]. It is necessary to deepen research in this field and expand the study to a greater number of individuals with different characteristics.

## 10. Considerations for Melatonin Supplementation

Exogenous melatonin has been investigated as a treatment for a number of different diseases; however, there are some doubts regarding its optimal dosage and bioavailability, despite its proven safety.

Melatonin is typically used in short-term sleep disorders or jet lag management, but its prolonged use should be approached with caution due to the limited long-term safety data. As a medicinal sleep aid, a typical single daily dose of 1–10 mg is considered standard, although the optimal dosing and administration route remain unclear for most indications [130,137,138]. The optimal dosage depends on factors such as age, weight, and disease severity [139]. Melatonin is commonly accessible in oral immediate-release and oral prolonged-release formulations [32,138]. It exhibits poor absorption across all formulations, with a bioavailability ranging from 2.5% to 33% and a protein binding rate of 60% in vitro. Significant hepatic metabolism occurs, particularly for oral formulations, due to a high hepatic first-pass effect [32,138]. Melatonin exhibits CYP450 metabolism (CYP1A2) [140].

Extensive research, both in animal and human studies, supports the safety of short-term melatonin usage, even at elevated doses. There is no indication of severe adverse effects resulting from exogenous melatonin intake. The absence of human studies demands caution among pregnant and breastfeeding women who are considering the use of exogenous melatonin. Additional research is also needed to assess the long-term safety profile of melatonin in children and adolescents [3,137].

### 10.1. The Therapeutic Potential of Melatonin in Treating Vascular Dysfunction

Melatonin has potential in treating vascular dysfunction due to its antioxidant, anti-inflammatory, and receptor-mediated actions. Melatonin has been shown in almost all studies to have beneficial effects on cardiovascular physiology and to protect the myocardium from injury following an ischemic heart attack, internal injury, or sepsis [16,113]. Heart arrhythmias and blood pressure can both benefit from melatonin. Melatonin should be investigated in many more comprehensive clinical trials to determine its effectiveness in treating a range of cardiovascular disorders because it is cheap and safe when taken in appropriate amounts. Furthermore, melatonin use in cardiovascular diseases is linked to a greater variability in its cardioprotective effects, according to certain clinical research [137,141]. Other than dosage and administration issues, previous failures may have been partially due to the use of young, healthy animals that eventually lacked various cardiovascular risk factors, comorbidities, and comedications—the characteristics of patients experiencing an acute myocardial infarction or undergoing cardiovascular surgery [142]. In light of the present setback, more carefully thought-out preclinical and clinical research is required to better define the cardiovascular benefits of melatonin [143].

Future research directions include understanding the roles of MT1 and MT2 receptors in vascular health, conducting dose–response and pharmacokinetic studies, comparing the efficacy of melatonin with other antioxidants and anti-inflammatory agents, conducting long-term clinical trials, exploring genetic and epigenetic factors in melatonin response variability, exploring melatonin analogs, integrating melatonin with lifestyle and dietary interventions, and evaluating the effects of melatonin in specific populations. These directions aim to provide evidence for comprehensive lifestyle-based treatment strategies that include melatonin supplementation, and tailor melatonin-based treatments for patients with specific comorbidities. The ultimate goal is to provide robust evidence for the long-term safety and efficacy of melatonin in preventing and treating vascular dysfunction.

### 10.2. Administration of Melatonin and Known Side Effects

Melatonin levels naturally rise in the evening, peak during the night, and fall in the early morning [18]. The timing of melatonin administration in studies is critical, as it can significantly impact its effectiveness and the potential side effects [144]. Aligning melatonin intake with the natural production cycle of the body enhances its benefits for sleep and circadian rhythm regulation, while reducing the risk of adverse effects. Understanding these temporal dynamics is essential, particularly when extrapolating animal research findings to human applications [14].

In humans, melatonin is typically administered in the evening or at night to align with the natural production of the body and to promote sleep. Evening administration helps to synchronize the internal clock with the desired sleep schedule, which is particularly useful in conditions such as jet lag or shift work disorder [145]. Nocturnal animals, such as mice, have a different circadian rhythm compared with humans. Their melatonin levels peak during their active night phase [146]. In studies involving mice, melatonin is usually given during their night phase to mirror the natural pattern; however, care must be taken to translate the findings from nocturnal animals to diurnal humans accurately. Administering melatonin during the day, when its levels are naturally low, can disrupt the diurnal rhythm [146]. This can lead to the desynchronization of internal clocks, leading to disturbed sleep patterns and other circadian-related disorders. Irregular melatonin levels can also affect mood, cognitive functions, and overall well-being, as these are closely tied to the sleep–wake cycle [147].

Melatonin is generally safe and well tolerated but it is important to be aware of the potential side effects in some individuals, especially when taken in high doses or for extended periods. Melatonin can cause daytime drowsiness, altered sleep patterns, gastrointestinal symptoms, headaches, mood changes, hormone effects, and interactions with medications. It can impair alertness and concentration; disrupt sleep patterns; cause nausea, stomach cramps, diarrhea, and headaches; and affect hormone levels, including estrogen and testosterone. It may also interact with blood thinners, immunosuppressants, and antidepressants, potentially affecting their effectiveness or increasing their side effects [148].

## 11. Conclusions

**Figure 5:**
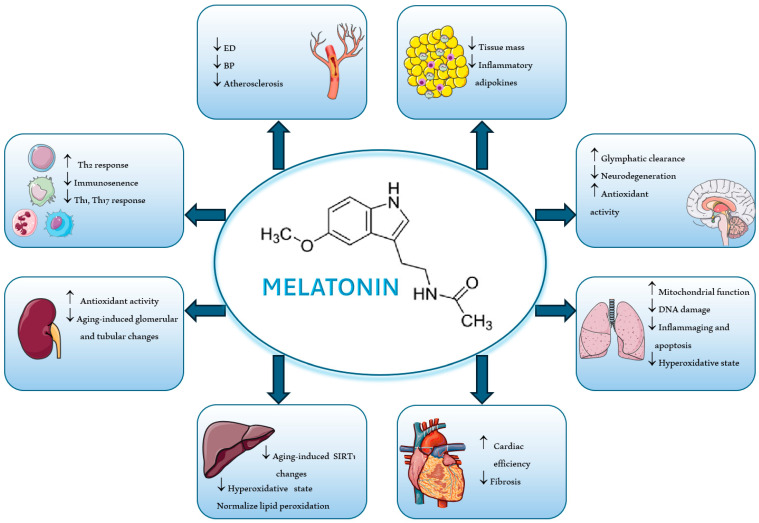
The beneficial potential of melatonin in different organs. BP, blood pressure; ED, endothelial dysfunction.

Cardiovascular diseases, such as hypertension and atherosclerosis, are widely associated with states of inflammation and oxidative stress, causing a decrease in the production of NO, a potent vasodilator and, therefore, endothelial dysfunction. Melatonin is a pleiotropic molecule, which, in addition to its regulatory function in the sleep–wake cycle, has proven to be a powerful antioxidant, through its radical scavenging properties, the activation of antioxidant enzymes, and the inhibition of pro-oxidant enzymes. Through mechanisms involving vasodilation, antioxidant activity, anti-inflammatory effects, blood pressure regulation, protection against ischemia-reperfusion injury, and lipid metabolism modulation, melatonin contributes significantly to cardiovascular health. In this way, melatonin emerges as a possible therapy to help in the treatment of different cardiovascular diseases. However, although there are many studies that point to the beneficial effects of melatonin supplementation, there are also some inconsistent results, meaning that more research is needed in order to draw conclusions about the effectiveness of this molecule. Ongoing research continues to unravel its complex interactions and therapeutic potential in the vascular system. A schematic diagram summarizing the key actions of melatonin in different organs is presented (Figure 5).
